# Standardizing effect size from linear regression models with log-transformed variables for meta-analysis

**DOI:** 10.1186/s12874-017-0322-8

**Published:** 2017-03-17

**Authors:** Miguel Rodríguez-Barranco, Aurelio Tobías, Daniel Redondo, Elena Molina-Portillo, María José Sánchez

**Affiliations:** 1 Andalusian School of Public Health (EASP), Campus Universitario de Cartuja, c/Cuesta del Observatorio 4, 18080 Granada, Spain; 2 Instituto de Investigación Biosanitaria ibs.GRANADA, University Hospitals of Granada/University of Granada, Granada, Spain; 3 CIBERESP, Madrid, Spain; 4 Institute of Environmental Assessment and Water Research (IDAEA), Spanish Council for Scientific Research (CSIC), Barcelona, Spain

**Keywords:** Meta-analysis, Systematic review, Log-transformation, Linear regression, Effect size, Regression coefficients

## Abstract

**Background:**

Meta-analysis is very useful to summarize the effect of a treatment or a risk factor for a given disease. Often studies report results based on log-transformed variables in order to achieve the principal assumptions of a linear regression model. If this is the case for some, but not all studies, the effects need to be homogenized.

**Methods:**

We derived a set of formulae to transform absolute changes into relative ones, and vice versa, to allow including all results in a meta-analysis. We applied our procedure to all possible combinations of log-transformed independent or dependent variables. We also evaluated it in a simulation based on two variables either normally or asymmetrically distributed.

**Results:**

In all the scenarios, and based on different change criteria, the effect size estimated by the derived set of formulae was equivalent to the real effect size. To avoid biased estimates of the effect, this procedure should be used with caution in the case of independent variables with asymmetric distributions that significantly differ from the normal distribution. We illustrate an application of this procedure by an application to a meta-analysis on the potential effects on neurodevelopment in children exposed to arsenic and manganese.

**Conclusions:**

The procedure proposed has been shown to be valid and capable of expressing the effect size of a linear regression model based on different change criteria in the variables. Homogenizing the results from different studies beforehand allows them to be combined in a meta-analysis, independently of whether the transformations had been performed on the dependent and/or independent variables.

**Electronic supplementary material:**

The online version of this article (doi:10.1186/s12874-017-0322-8) contains supplementary material, which is available to authorized users.

## Background

A meta-analysis is a systematic review of the literature that uses statistical methods to combine the results of two or more eligible studies [1]. It is useful because it provides a more accurate effect estimate by identifying clinically important effects, which, because of their size, may not have been detected in the primary studies. Furthermore, with meta-analyses it is possible to obtain a higher level of precision thanks to a larger sample size.

The type of measurement used to calculate effect size depends on the estimators used in the studies included in the meta-analysis [2]. Therefore, one of the possible limitations in a meta-analysis is that published studies report results that were obtained through different analytical approaches and measures of association. When performing a meta-analysis of an effect size estimated with linear regression models, this limitation can be (at least to a certain extent) overcome by using different transformations. Consequently, variables in linear regression models are usually transformed to achieve the principal assumptions of i) linearity of the relationship, ii) independence of the residual values, iii) homoscedasticity (constant variance) of the residuals, and iv) normal distribution of the residuals [3, 4]. Depending on the transformation applied in each case (natural logarithm, base 2 logarithm, base 10, etc.), and whether it is performed on an independent variable, dependent variable or both, the regression coefficient is interpreted differently [3, 4].

**Fig. 1:**
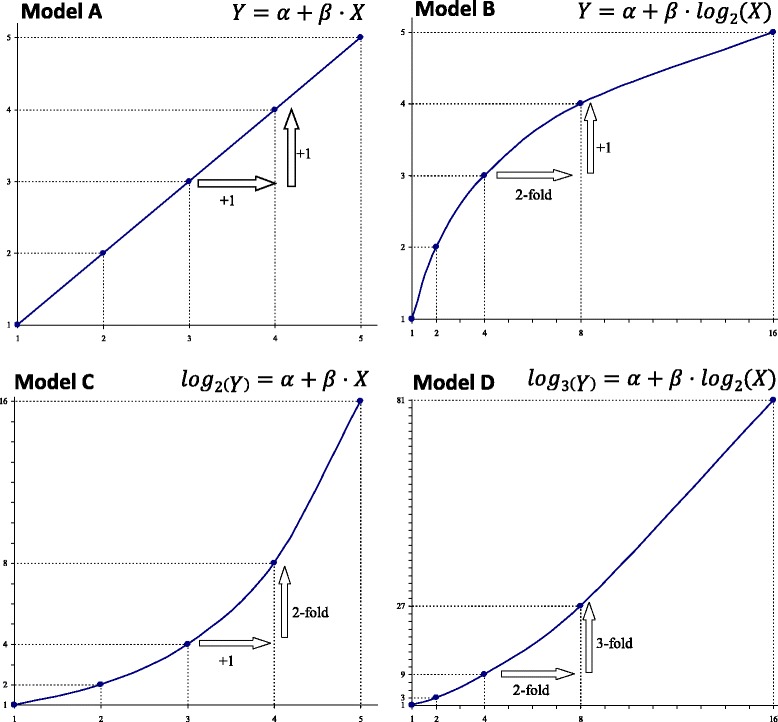
Relationship between X and Y changes in a linear model with logarithmic transformations

In a linear relationship between two untransformed variables, we quantify the absolute change in one of them by an absolute change in the other. However, when a variable is transformed logarithmically, the absolute variation in the logarithm equals a relative variation of the original variable (Fig. 1) For example, an increase of one unit in the logarithmically transformed variable is equivalent to multiplying the original variable by the base of the logarithm used. The existence of these transformations will, therefore, affect the interpretation of the effect size.

Thus, before performing a meta-analysis, some pre-processing procedure to homogenize the magnitude of effect observed in each study is required. This means that recalculating each effect to express it as a change in the dependent variable that corresponds to the same change in the independent variable is required. These changes, depending on the absence or presence of logarithmic transformation, can be expressed in either absolute or relative terms. Recent studies have applied a methodology to standardize the results of linear regression models through the logarithmic transformation of the independent variable in different bases for their inclusion in a meta-analysis [5].

This study aimed to develop a set of formulae to express results from linear regression models with different log-transformations of independent and/or dependent variables as the same effect size to be included in a meta-analysis.

## Methods

The linear regression model, a commonly used statistic tool, establishes a linear relation between two variables and estimates its association. The simplest linear regression models can be written as

$$ Y=\alpha +\beta \cdotp X+\varepsilon $$

where *α* is the ordinate at the origin of the straight line that relates *X* to *Y*, *β* is the slope of the straight line that relates *X* to *Y*, and *ε* is the random error.

If we call $\widehat{\alpha}$ and $\widehat{\beta}$ the estimates of *α* and *β* by the least squares approach (that is, minimizing the squared distance between the estimation and the observed value), then we can write the following equation:

$$ \widehat{Y}=\widehat{\alpha}+\widehat{\beta}\cdotp X $$

The estimator $\widehat{\beta}$ measures the strength of association between *X* and *Y*, as this represents the absolute change in the mean of *Y* for an increase of one unit in *X*. However, the meaning of $\widehat{\beta}$ is not as intuitive when variables are transformed.

All possible regression models with all possible combinations of log-transformations for the dependent or independent variables were considered. Thus, the following models were formulated: (i) no transformation (model A), (ii) only the independent variable transformed (model B), (iii) only the dependent variable transformed (model C), and (iv) both the dependent and independent variables transformed (model D) (see Fig. 1). Log-transformations were expressed in a general base *a* for the dependent variable and in base *b* for the independent variable. Absolute change in a variable was set as *c* units and relative change was considered to be a ratio *k* between values.

Table 1 shows all possible scenarios based on the model (A to D) considered and the combination of absolute or relative change in the dependent or independent variables. The effect size and the 95% confidence interval (CI) are shown in each cell. The diagonal line in the table indicates the expression of the effect size and the 95% CI that directly corresponds to that particular model [3]. The other formulae proposed express effects that differ from those obtained directly from the model. The formulae stem from basic transformations to express absolute changes as relative changes and vice versa:

**Table 1:** Expressions of effect size and the 95% confidence interval estimation for each model and set of change criteria

	Model A $\widehat{Y}=\widehat{\alpha}+\widehat{\beta}\cdotp X$	Model B $\widehat{Y}=\widehat{\alpha}+\widehat{\beta}\cdotp { \log}_b(X)$	Model C $\widehat{{ \log}_a(Y)}=\widehat{\alpha}+\widehat{\beta}\cdotp X$	Model D $\widehat{{ \log}_a(Y)}=\widehat{\alpha}+\widehat{\beta}\cdotp { \log}_b(X)$
Absolute change in *Y* for an absolute change of *c* units in *X*	$$ \begin{array}{c}\hfill c\cdotp \widehat{\beta}\hfill \\ {}\hfill \mathrm{c}\cdotp \left[\widehat{\beta}\pm 1.96\cdotp \mathrm{se}\left(\widehat{\beta}\right)\right]\hfill \end{array} $$ (5), (6)	$$ \begin{array}{c}\hfill { \log}_b\left(1+\frac{c}{E\left[ X\right]}\right)\cdotp \widehat{\beta}\hfill \\ {}\hfill { \log}_b\left(1+\frac{c}{E\left[ X\right]}\right)\cdotp \left[\widehat{\beta}\pm 1.96\cdotp \mathrm{se}\left(\widehat{\beta}\right)\right]\hfill \end{array} $$ (2), (13), (14)	$$ \begin{array}{c}\hfill \left({a}^{c\cdotp \widehat{\beta}}-1\right)\cdotp E\left[ Y\right]\hfill \\ {}\hfill \left({a}^{c\cdotp \left[\widehat{\beta}\pm 1.96\cdotp \mathrm{se}\left(\widehat{\beta}\right)\right]}-1\right)\cdotp E\left[ Y\right]\hfill \end{array} $$ (4), (22), (23)	$$ \begin{array}{c}\hfill \left({a}^{\log_b\left(1+\frac{c}{E\left[ X\right]}\right)\cdotp \widehat{\beta}}-1\right)\cdotp E\left[ Y\right]\hfill \\ {}\hfill \left({a}^{\log_b\left(1+\frac{c}{E\left[ X\right]}\right)\cdotp \left[\widehat{\beta}\pm 1.96\cdotp \mathrm{se}\left(\widehat{\beta}\right)\right]}-1\right)\cdotp E\left[ Y\right]\hfill \end{array} $$ (2), (33), (34)
Absolute change in *Y* for a relative change of *k* times in *X*	$$ \begin{array}{c}\hfill \left( k-1\right)\cdotp \mathrm{E}\left[ X\right]\cdotp \widehat{\beta}\hfill \\ {}\hfill \left( k-1\right)\cdotp \mathrm{E}\left[ X\right]\cdotp \left[\widehat{\beta}\pm 1.96\cdotp \mathrm{se}\left(\widehat{\beta}\right)\right]\hfill \end{array} $$ (1), (5), (6)	$$ \begin{array}{c}\hfill { \log}_b(k)\cdotp \widehat{\beta}\hfill \\ {}\hfill { \log}_b(k)\cdotp \left[\widehat{\beta}\pm 1.96\cdotp \mathrm{se}\left(\widehat{\beta}\right)\right]\hfill \end{array} $$ (13), (14)	$$ \begin{array}{c}\hfill \left({a}^{\left( k-1\right)\cdotp \mathrm{E}\left[ X\right]\cdotp \widehat{\beta}}-1\right)\cdotp E\left[ Y\right]\hfill \\ {}\hfill \left({a}^{\left( k-1\right)\cdotp \mathrm{E}\left[ X\right]\cdotp \left[\widehat{\beta}\pm 1.96\cdotp \mathrm{se}\left(\widehat{\beta}\right)\right]}-1\right)\cdotp E\left[ Y\right]\hfill \end{array} $$ (1), (4), (26)	$$ \begin{array}{c}\hfill \left({a}^{\log_b(k)\cdotp \widehat{\beta}}-1\right)\cdotp E\left[ Y\right]\hfill \\ {}\hfill \left({a}^{\log_b(k)\cdotp \left[\widehat{\beta}\pm 1.96\cdotp \mathrm{se}\left(\widehat{\beta}\right)\right]}-1\right)\cdotp E\left[ Y\right]\hfill \end{array} $$ (4), (29), (30)
Relative change in *Y* for an absolute change of *c* units in *X*	$$ \begin{array}{c}\hfill 1+\frac{c\cdotp \widehat{\beta}}{E\left[ Y\right]}\hfill \\ {}\hfill \frac{c}{E\left[ Y\right]}\left\{\frac{E\left[ Y\right]}{c}+\widehat{\beta}\pm 1.96\cdotp se\left(\widehat{\beta}\right)\right\}\hfill \end{array} $$ (3), (5), (6)	$$ \begin{array}{c}\hfill 1+\frac{{ \log}_b\left(1+\frac{c}{E\left[ X\right]}\right)\cdotp \widehat{\beta}}{E\left[ Y\right]}\hfill \\ {}\hfill \frac{{ \log}_b\left(1+\frac{c}{E\left[ X\right]}\right)}{E\left[ Y\right]}\left\{\frac{E\left[ Y\right]}{{ \log}_b\left(1+\frac{c}{E\left[ X\right]}\right)}+\widehat{\beta}\pm 1.96\cdotp se\left(\widehat{\beta}\right)\right\}\hfill \end{array} $$ (2), (17), (19)	$$ \begin{array}{c}\hfill {a}^{c\cdotp \widehat{\beta}}\hfill \\ {}\hfill {a}^{c\cdotp \left[\widehat{\beta}\pm 1.96\cdotp \mathrm{se}\left(\widehat{\beta}\right)\right]}\hfill \end{array} $$ (22), (23)	$$ \begin{array}{c}\hfill {a}^{\log_b\left(1+\frac{c}{E\left[ X\right]}\right)\cdotp \widehat{\beta}}\hfill \\ {}\hfill {a}^{\log_b\left(1+\frac{c}{E\left[ X\right]}\right)\cdotp \left[\widehat{\beta}\pm 1.96\cdotp \mathrm{se}\left(\widehat{\beta}\right)\right]}\hfill \end{array} $$ (2), (29), (30)
Relative change in *Y* for a relative change of *k* times in *X*	$$ \begin{array}{c}\hfill 1+\frac{\left( k-1\right)\cdotp \mathrm{E}\left[ X\right]\cdotp \widehat{\beta}}{E\left[ Y\right]}\hfill \\ {}\hfill \frac{\left( k-1\right)\cdotp \mathrm{E}\left[ X\right]}{E\left[ Y\right]}\left\{\frac{E\left[ Y\right]}{\left( k-1\right)\cdotp \mathrm{E}\left[ X\right]}+\widehat{\beta}\pm 1.96\cdotp se\left(\widehat{\beta}\right)\right\}\hfill \end{array} $$ (1), (3), (10)	$$ \begin{array}{c}\hfill 1+\frac{{ \log}_b(k)\cdotp \widehat{\beta}}{E\left[ Y\right]}\hfill \\ {}\hfill \frac{{ \log}_b(k)}{E\left[ Y\right]}\left\{\frac{E\left[ Y\right]}{{ \log}_b(k)}+\widehat{\beta}\pm 1.96\cdotp se\left(\widehat{\beta}\right)\right\}\hfill \end{array} $$ (3), (13), (14)	$$ \begin{array}{c}\hfill {a}^{\left( k-1\right)\cdotp \mathrm{E}\left[ X\right]\cdotp \widehat{\beta}}\hfill \\ {}\hfill {a}^{\left( k-1\right)\cdotp \mathrm{E}\left[ X\right]\cdotp \left[\widehat{\beta}\pm 1.96\cdotp \mathrm{se}\left(\widehat{\beta}\right)\right]}\hfill \end{array} $$ (1), (22), (23)	$$ \begin{array}{c}\hfill {a}^{\log_b(k)\cdotp \widehat{\beta}}\hfill \\ {}\hfill {a}^{\log_b(k)\cdotp \left[\widehat{\beta}\pm 1.96\cdotp \mathrm{se}\left(\widehat{\beta}\right)\right]}\hfill \end{array} $$ (29), (30)

Note: Numbers in the bottom of cells indicate equations involved in derivation. Formulae (1, 2, 3 and 4) are found in the main text and (5) to (34) are found in Additional file 1

- Equivalent absolute change *c* in X for a relative change *k* in X.

The objective was to obtain the equivalent of an absolute change of *c* units in the independent variable for a relative change equal to *k* in the independent variable. For that purpose, we approximated the absolute change that would occur in the independent variable as a relative change equal to *k* in the mean of its distribution.

1 $$ c=\left( k-1\right)\cdotp E\left[ X\right] $$

- Equivalent relative change *k* in X for an absolute change *c* in X.

Similarly, the relative change corresponding to an absolute change of *c* units in the independent variable was approximated as follows:

2 $$ k=1+ c/ E\left[ X\right] $$

- Equivalent relative change *k′* in Y for an absolute change *c′* in Y.

In the case of the non-transformed dependent variable, the regression model provided the absolute change in Y (*c′* = *c · β*) for an absolute or relative change in X. Analogously to Eq. (2), the following approximation was performed to obtain the equivalent relative change in Y:

3 $$ {k}^{\prime }=1+{c}^{\prime }/ E\left[ Y\right]=1+ c\cdotp \beta / E\left[ Y\right] $$

- Equivalent absolute change *c′* in Y for a relative change *k′* in Y.

When the dependent variable was log-transformed, the model provided the relative change in Y(*k*′ = *a* ^*c* · *β*^) for an absolute or relative change in X. Analogously to Eq. (1), the following approximation was performed to obtain an equivalent absolute change in Y:

4 $$ {c}^{\prime }=\left({k}^{\prime }-1\right)\cdotp E\left[ Y\right]=\left({a}^{c\cdotp \beta}-1\right)\cdotp E\left[ Y\right] $$

Thus, with these transformations the formulae in Table 1, based on the combinations of the different models and effect expressions, were obtained (see Additional file 1 for derivations).

## Simulation

To evaluate the error resulting from the approximations, we built each of the four models and ran simulations. A database with random samples of 500 values from standard distributions of probability (normal and lognormal distributions) was generated. Natural logarithm transformed and untransformed variables were used, and the real values of the regression coefficient and the standard errors from each model A to D were estimated (Tables 2, 3, 4 and 5). Next, the formulae in Table 1 were applied to these values to obtain the effect size from the different change expressions. The simulation was performed for four different scenarios, depending on the following distributions of dependent and independent variables: when the two variables are normally distributed, when the two variables have asymmetric distributions, and when one of the variables has a normal distribution and the other has an asymmetric distribution. In all cases, the mean value of the dependent variable was equal to 50 and the mean value of the independent value was equal to 10. The variables were generated in such a way that the increase of a unit in X was associated with an increase of approximately one unit in Y.

**Table 2:** Simulation results when X and Y are normally distributed

		Model A $\widehat{Y}=\widehat{\alpha}+\widehat{\beta}\cdotp X$	Model B $\widehat{Y}=\widehat{\alpha}+\widehat{\beta}\cdotp { \log}_b(X)$	Model C $\widehat{{ \log}_a(Y)}=\widehat{\alpha}+\widehat{\beta}\cdotp X$	Model D $\widehat{{ \log}_a(Y)}=\widehat{\alpha}+\widehat{\beta}\cdotp { \log}_b(X)$
Beta-hat coefficient and standard error from regression model		$\widehat{\beta}$ = 0.995 se($\widehat{\beta}$) = 0.054	$\widehat{\beta}$ = 9.587 se($\widehat{\beta}$) = 0.520	$\widehat{\beta}$ = 0.020 se($\widehat{\beta}$) = 0.001	$\widehat{\beta}$ = 0.193 se($\widehat{\beta}$) = 0.011
Absolute change in *Y* for an absolute change of *c* units in *X*	Effect size	0.995	0.914	1.006	0.928
	95% CI	(0.889–1.101)	(0.817–1.011)	(0.895–1.118)	(0.827–1.029)
Absolute change in *Y* for a relative change of *k* times in *X*	Effect size	0.995	0.914	1.006	0.928
	95% CI	(0.889–1.101)	(0.817–1.011)	(0.895–1.118)	(0.827–1.029)
Relative change in *Y* for an absolute change of *c* units in *X*	Effect size	1.0199	1.0183	1.0201	1.0186
	95% CI	(1.0178–1.0220)	(1.0163–1.0202)	(1.0179–1.0224)	(1.0165–1.0206)
Relative change in *Y* for a relative change of *k* times in *X*	Effect size	1.0199	1.0183	1.0201	1.0186
	95% CI	(1.0178–1.0220)	(1.0163–1.0202)	(1.0179–1.0224)	(1.0165–1.0206)

Note: *c* = 1 and *k* = 1.1

**Table 3:** Simulation results when X and Y have an asymmetric distribution

		Model A $\widehat{Y}=\widehat{\alpha}+\widehat{\beta}\cdotp X$	Model B $\widehat{Y}=\widehat{\alpha}+\widehat{\beta}\cdotp { \log}_b(X)$	Model C $\widehat{{ \log}_a(Y)}=\widehat{\alpha}+\widehat{\beta}\cdotp X$	Model D $\widehat{{ \log}_a(Y)}=\widehat{\alpha}+\widehat{\beta}\cdotp { \log}_b(X)$
Beta-hat coefficient and standard error from regression model		$\widehat{\beta}$ = 0.997 se($\widehat{\beta}$) = 0.009	$\widehat{\beta}$ = 6.071 se($\widehat{\beta}$) = 0.213	$\widehat{\beta}$ = 0.018 se($\widehat{\beta}$) = 0.0002	$\widehat{\beta}$ = 0.115 se($\widehat{\beta}$) = 0.003
Absolute change in *Y* for an absolute change of *c* units in *X*	Effect size	0.997	0.579	0.894	0.551
	95% CI	(0.980–1.014)	(0.539–0.618)	(0.874–0.915)	(0.518–0.584)
Absolute change in *Y* for a relative change of *k* times in *X*	Effect size	0.997	0.579	0.894	0.551
	95% CI	(0.980–1.014)	(0.539–0.618)	(0.874–0.915)	(0.518–0.584)
Relative change in *Y* for an absolute change of *c* units in *X*	Effect size	1.0199	1.0116	1.0179	1.0110
	95% CI	(1.0196–1.0203)	(1.0108–1.0124)	(1.0175–1.0183)	(1.0100–1.0117)
Relative change in *Y* for a relative change of *k* times in *X*	Effect size	1.0199	1.0116	1.0179	1.0110
	95% CI	(1.0196–1.0203)	(1.0108–1.0124)	(1.0175–1.0183)	(1.0100–1.0117)

Note: *c* = 1 and *k* = 1.1

**Table 4:** Simulation results when Y has an asymmetric distribution

		Model A $\widehat{Y}=\widehat{\alpha}+\widehat{\beta}\cdotp X$	Model B $\widehat{Y}=\widehat{\alpha}+\widehat{\beta}\cdotp { \log}_b(X)$	Model C $\widehat{{ \log}_a(Y)}=\widehat{\alpha}+\widehat{\beta}\cdotp X$	Model D $\widehat{{ \log}_a(Y)}=\widehat{\alpha}+\widehat{\beta}\cdotp { \log}_b(X)$
Beta-hat coefficient and standard error from regression model		$\widehat{\beta}$ = 0.625 se($\widehat{\beta}$) = 0.254	$\widehat{\beta}$ = 5.834 se($\widehat{\beta}$) = 2.441	$\widehat{\beta}$ = 0.011 se($\widehat{\beta}$) = 0.005	$\widehat{\beta}$ = 0.103 se($\widehat{\beta}$) = 0.044
Absolute change in *Y* for an absolute change of *c* units in *X*	Effect size	0.625	0.557	0.551	0.493
	95% CI	(0.128–1.122)	(0.101–1.013)	(0.100–1.006)	(0.080–0.909)
Absolute change in *Y* for a relative change of *k* times in *X*	Effect size	0.625	0.557	0.551	0.493
	95% CI	(0.128–1.122)	(0.101–1.013)	(0.100–1.006)	(0.080–0.909)
Relative change in *Y* for an absolute change of *c* units in *X*	Effect size	1.0125	1.0111	1.0110	1.0099
	95% CI	(1.0026–1.0224)	(1.0020–1.0203)	(1.0020–1.0201)	(1.0016–1.0182)
Relative change in *Y* for a relative change of *k* times in *X*	Effect size	1.0125	1.0111	1.0110	1.0099
	95% CI	(1.0026–1.0224)	(1.0020–1.0203)	(1.0020–1.0201)	(1.0016–1.0182)

Note: *c* = 1 and *k* = 1.1

**Table 5:** Simulation results when X has an asymmetric distribution

		Model A $\widehat{Y}=\widehat{\alpha}+\widehat{\beta}\cdotp X$	Model B $\widehat{Y}=\widehat{\alpha}+\widehat{\beta}\cdotp { \log}_b(X)$	Model C $\widehat{{ \log}_a(Y)}=\widehat{\alpha}+\widehat{\beta}\cdotp X$	Model D $\widehat{{ \log}_a(Y)}=\widehat{\alpha}+\widehat{\beta}\cdotp { \log}_b(X)$
Beta-hat coefficient and standard error from regression model		$\widehat{\beta}$ = 0.288 se($\widehat{\beta}$) = 0.015	$\widehat{\beta}$ = 1.517 se($\widehat{\beta}$) = 0.133	$\widehat{\beta}$ = 0.005 se($\widehat{\beta}$) = 0.0003	$\widehat{\beta}$ = 0.028 se($\widehat{\beta}$) = 0.003
Absolute change in *Y* for an absolute change of *c* units in *X*	Effect size	0.288	0.145	0.263	0.133
	95% CI	(0.259–0.317)	(0.120–0.169)	(0.236–0.291)	(0.109–0.156)
Absolute change in *Y* for a relative change of *k* times in *X*	Effect size	0.288	0.145	0.263	0.133
	95% CI	(0.259–0.317)	(0.120–0.169)	(0.236–0.291)	(0.109–0.156)
Relative change in *Y* for an absolute change of *c* units in *X*	Effect size	1.0058	1.0029	1.0053	1.0027
	95% CI	(1.0052–1.0063)	(1.0024–1.0034)	(1.0047–1.0058)	(1.0022–1.0031)
Relative change in *Y* for a relative change of *k* times in *X*	Effect size	1.0058	1.0029	1.0053	1.0027
	95% CI	(1.0052–1.0063)	(1.0024–1.0034)	(1.0047–1.0058)	(1.0022–1.0031)

Note: *c* = 1 and *k* = 1.1

For the simulations, the parameters *c* = 1 and *k* = 1.1 were fixed to reflect the effect of an absolute change in one unit or a relative change of 10% in the independent variable (equivalent to one unit given that the mean value of X is 10). Tables 2, 3, 4 and 5 show the results of the simulations. The diagonal positions in these tables correspond to the real effect size, which is obtained from the regression coefficient and the standard error of the specific model. The remainder of the values in each row represents the estimated effect size when using the formulae.

## Results

In all of the scenarios, the effect size estimated from the formulae, based on different change criteria, was equivalent to the real effect size. In the model without transformation (model A), the variation of a unit in X is associated with a variation of 0.995 units in Y. When the formula to express a variation of X in relative terms was applied (i.e. an increase of 10% in X as equivalent to one unit), the same result (beta = 0.995) was produced. On the other hand, the estimated effect on Y in relative terms was 1.0199, i.e. a variation of 1.99%. Given that the mean value of Y is 50 units, that variation is equivalent to an increase of 0.995 units, which is equal to the real effect observed (Table 1).

For the other models, the result was the same. In model B, the real absolute change was 0.914, whereas the estimated relative change was 1.0183 (1.83% or 0.914 units), while in model C, the real relative change of 1.0201 equaled the estimated absolute change of 1.006 units, and in model D the real relative change of 1.0186 was equivalent to the estimated absolute change of 0.928. This equivalence, based on the different distributions of variables X and Y (Tables 2, 3, 4 and 5), was maintained in all the scenarios contemplated.

The variation in effect size between the various models differed depending on the shape of the distribution of variables. For the relationship between normally distributed variables, the range of variation in the absolute effect was 0.914 to 1.006, and 1.0183 to 1.0201 in the relative effect. When the independent variable only was skewed, the absolute effect varied between 0.133 and 0.288 and the relative effect between 1.0027 and 1.0058, while when the dependent variable only was skewed, the absolute effect varied between 0.493 and 0.625 and the relative effect between 1.0099 and 1.0125, and when both variables were skewed, the absolute effect varied between 0.551 and 0.997 and the relative effect between 1.0110 and 1.0199.

## Empirical example

The method proposed in this study was successfully used in a systematic review that performed a meta-analysis on the potential effects on neurodevelopment in children exposed to arsenic (As) and manganese (Mn) [5]. Additional details on the search strategy, target population, inclusion and exclusion criteria, and assessment of methodological quality have been previously reported [5]. Studies that evaluated neurodevelopment using the same scale (the Wechsler scale [6]) and linear regression techniques to estimate the effect were included in a meta-analysis. Three independent meta-analyses were performed as per the metallic element studied and the sample type: arsenic in urine (five studies included) [7–11], arsenic in drinking water (four studies) [8–11] and manganese in hair (four studies) [12–15]. To assess the association of metal exposure with the full-scale intelligence quotient (IQ) from the Wechsler scale, all the studies used model A (without transformations) or model B (with log-transformed independent variable), with metal exposure as the independent variable and intelligent quotient as the dependent variable. Table 6 shows the type of transformation on X, original regression coefficients and transformed effect sizes, in accordance with the formulae proposed in this study. All effect sizes were expressed as the absolute change in the dependent variable (Y) for an increase of 50% in the independent variable (X), which is equivalent to a coefficient *k* = 1.5. Thus, transformed effect sizes express the absolute change in the intelligence quotient for a 50% increase in the metal levels.

**Table 6:** Original regression coefficients and transformed effect size for studies included in the meta-analysis

Author (Year)	Mean of X	Units	Transf. on X	β	SE(β)	θ	SE(θ)
As in urine							
Hamadani (2011)-Girls [7]		μg/L	Ln	−1.40	0.66	−0.57	0.27
Hamadani (2011)-Boys [7]		μg/L	Ln	0.70	0.56	0.28	0.23
Rocha-Amador (2007) [8]		μg/gr crea	Ln	−5.72	1.93	−2.32	0.78
von Ehrenstein (2007) [9]	78	μg/L	None	−0.0007	0.0008	−0.03	0.03
Wasserman (2007) [10]		μg/gr crea	Ln	−1.78	1.42	−0.72	0.58
Wasserman (2004) [11]		μg/gr crea	Ln	−2.90	1.71	−1.18	0.69
As in water							
Rocha-Amador (2007) [8]		μg/L	Ln	−6.15	1.87	−2.49	0.76
von Ehrenstein (2007) [9]	147	μg/L	None	−0.0002	0.0004	−0.01	0.03
Wasserman (2007) [10]		μg/L	Ln	−1.06	0.57	−0.43	0.23
Wasserman (2004) [11]		μg/L	Ln	−1.64	0.64	−0.66	0.26
Mn in hair							
Bouchard (2011) [12]		μg/g	log10	−3.30	1.43	−0.58	0.25
Menezes-Filho (2011) [13]		μg/g	log10	−5.78	2.84	−1.02	0.50
Riojas-Rodríguez (2010) [14]	6.35	μg/g	None	−0.20	0.11	−0.64	0.36
Wright (2006) [15]	0.47	μg/g	None	−10.00	5.00	−2.35	1.18

*As*, arsenic, *Mn* manganese, *θ* transformed effect size for *k* = 1.5, *Ln* natural logarithm, *log10* base 10 logarithm, *gr crea* grams of creatinine

For example, results from Rocha-Amador in the meta-analysis of As in urine are from a regression model with natural logarithmic transformation of the independent variable (model B). To obtain the absolute change in the outcome for a relative increase of 1.5 times in the exposure, we apply the formulae in model B for that scenario (see Additional file 1, formulae (13) and (14)):

$$ \begin{array}{c}\hfill { \log}_b(k)\cdotp \beta = \ln (1.5)\cdotp \left(-5.72\right)=-2.32\hfill \\ {}\hfill { \log}_b(k)\cdotp \left[\beta \pm 1.96\cdotp se\left(\beta \right)\right]= \ln (1.5)\cdotp \left(-5.72\pm 1.96\cdotp 1.93\right)=-2.32\pm 1.53\hfill \end{array} $$

To obtain the equivalent effect size from von Ehrenstein’s results (which used model A without transformation) formulae (7) and (8) from Additional file 1 must be used:

$$ \begin{array}{c}\hfill \left( k-1\right)\cdotp \mathrm{E}(X)\cdotp \upbeta =0.5\cdotp 78\cdotp \left(-0.0007\right)=-0.03\hfill \\ {}\hfill \left( k-1\right)\cdotp \mathrm{E}(X)\cdotp \left[\upbeta \pm 1.96\cdotp \mathrm{se}\left(\upbeta \right)\right]=0.5\cdotp 78\cdotp \left(-0.0007\pm 1.96\cdotp 0.0008\right)=-0.03\pm 0.06\hfill \end{array} $$

The results of the meta-analysis suggested that for every 50% increase in arsenic levels (either in urine or in regular drinking water) there could be an approximately 0.5 decrease in the IQ of children aged 5–15 years. Moreover, a 50% increase in manganese levels in hair would be associated with a decrease of 0.7 points in the IQ of children aged 6–13 years [5].

This approach allowed the results from regression models using different formulations to be combined, and, thus obtain a pooled measure of association that included all available results.

## Discussion

To establish causality, well-conducted and free-of-bias systematic reviews that include a meta-analysis have been proposed as the epidemiological design at the top rank of the evidence-based medicine pyramid [16]. However, the main bias in such design is publication bias and while there are statistical methods that can be used to study the presence of this error, it cannot be controlled [17].

Another problem in meta-analyses is the difficulty of including all the studies dealing with the research topic, either because of a specific transformation performed on the variables of the model or because the effect measurements in said study were not relevant to the research question. When all studies on a specific topic cannot be included, the meta-analysis loses external validity. This difficulty would be solved if it were possible to access the original data (not only the results) that the authors had amassed. However, in almost all cases, accessing this kind of information is practically impossible.

An alternative would be to contact the author of the published study and request the results that were obtained from the original data but which do not appear in the publication. Occasionally this strategy provides a way to access the data required for the study to be included in the meta-analysis. However, such efforts are generally not successful, as positive responses are rare; particularly if the study had been conducted several years beforehand.

On the other hand, there are other initiatives that allow access to anonymized original data obtained in other studies. A relevant example of this is the data-sharing policy of the BMJ journals [18]. In fact, after 2013, the publication of the results from any clinical trial on drugs or medical devices requires the authors to make the relevant patient-level data available (on reasonable request) to other researchers.

In the absence of this type of strategy being consolidated and expanded, there is the urgent need to develop procedures that can be used to standardize results obtained with different methodological approaches so that they can then be validly combined in a meta-analysis. Such procedures would optimize meta-analyses as they would make it possible to include a maximum number of results, even when the analyses carried out were not identical. This would not only increase the statistical potential of the meta-analysis, but would also reduce the risk of any selection bias that might occur if some of the studies identified in the systematic review had to be excluded.

This study proposes a procedure to homogenize the estimated effect sizes with linear regression models that use different transformations of dependent and/or independent variables. The application of these transformations to express all the effect sizes based on the same change criterion enables the results from studies that have built their regression models with different transformations to estimate the effect to be combined. Furthermore, the generalization of the method also allows the effect size to be recalculated, independent of the logarithm base applied in the transformation. Simply reflecting the same change in the independent variable is all that is required.

The simulation results showed that this procedure provided an estimation of the effect that was equal to that obtained with the original model. Moreover, the approximation was not affected by the form of the distribution of the variables. Nevertheless, it is also important to compare the effects of the four models since, from a practical perspective, this procedure will be used to compare the results of different regression models.

As can be observed in the simulation, the effect estimate obtained by using a model without transformations is not the same as that obtained with a model that uses some type of transformation. In other words, if an author presents the result of a model with the log-transformed dependent variable and we then apply the procedure described to recalculate the effect based on a model without transformation, we would not obtain the same result as the author would from their own data in a model without transformation.

This limitation can produce a certain degree of bias in the effect estimate. Based on the simulation results, the size of this bias basically depends on the symmetry of the independent variable (X). When X and Y have a normal distribution, the variation of the effect size in regard to model A is, at most, 8%. When Y has an asymmetric distribution and X a normal distribution, the variation is approximately 10%. However, when the independent variable is asymmetric, the bias can be as high as 50% of the value of the effect estimated with model A.

To apply this model, the standard should be regarded as the most generalized model of all the results, and then the effect should be transformed for those results that use a different model. To apply the proposed formulae featured, an Excel spreadsheet is available as Additional file 2.

## Conclusions

In conclusion, the method proposed in this study was shown to be valid and capable of expressing the effect size of a linear regression model consistent with different change criteria in the variables involved. The previous homogenization of the results from different studies allows them to be combined in a meta-analysis, independent of the transformations performed on the dependent and/or independent variables. However, in order to avoid biased effect estimates, this procedure should be used with caution in the case of independent variables with asymmetric distributions that significantly differ from normal ones.

## Additional files

Additional file 1: Derivation of formulae in Table 1. (DOCX 19 kb)
Additional file 2: Excel template to transform original effect size using the proposed formulae. (XLSX 19 kb)

## Abbreviations

As: Arsenic
CI: Confidence interval
IQ: Intelligent quotient
Mn: Manganese
