# Erratum to: Standardizing effect size from linear regression models with log-transformed variables for meta-analysis

**DOI:** 10.1186/s12874-017-0365-x

**Published:** 2017-06-19

**Authors:** Miguel Rodríguez-Barranco, Aurelio Tobías, Daniel Redondo, Elena Molina-Portillo, María José Sánchez

**Affiliations:** 1 Andalusian School of Public Health (EASP), Campus Universitario de Cartuja, c/Cuesta del Observatorio 4, 18080 Granada, Spain; 2 Instituto de Investigación Biosanitaria ibs.GRANADA, University Hospitals of Granada/ University of Granada, Granada, Spain; 3 CIBERESP, Madrid, Spain; 4 Institute of Environmental Assessment and Water Research (IDAEA), Spanish Council for Scientific Research (CSIC), Barcelona, Spain

## Erratum

Since the publication of our article [1], it has come to our attention that the legend to Fig. 1 did not acknowledge that this figure had been adapted from a published source. The legend to Fig. 1 should read as follows:

Relationship between X and Y changes in a linear model with logarithmic transformations. Adapted with permission from eFigure 1 in Barrera-Gómez J and Basagaña X, Models with Transformed Variables: Interpretation and Software. *Epidemiology*. 2015; 26 (2): e16–17, http://journals.lww.com/epidem/Fulltext/2015/03000/Models_with_Transformed_Variables__Interpretation.27.aspx, copyright © 2015 Wolters Kluwer Health Inc. [3].”
