# Supplementary material for: Standardizing effect size from linear regression models with log-transformed variables for meta-analysis
Source: BMC Med Res Methodol. 2017 Mar 17;17:44. doi: 10.1186/s12874-017-0322-8 (PMC5356327; doi:10.1186/s12874-017-0322-8)
Supplement: Additional file 1: — Derivation of formulae in Table 1. (DOCX 19 kb) [file 12874_2017_322_MOESM1_ESM.docx]

**Additional file 1.** Derivation of formulas in Table 1

**Model A**

- Absolute change in *Y* for an absolute change of *c* units in *X*

Directly from the model, the expression of effect size and the 95% CI are provided for (5) and (6):

$c\cdot\beta$ (5)

$c\cdot[\beta\pm1.96\cdot se(\beta)]$ (6)

- Absolute change in *Y* for a relative change of *k* times in *X*

Replacing *c* in (5) and (6) in accordance with (1):

$(k-1)\cdot E[X]\cdot\beta$ (7)

$(k-1)\cdot E[X]\cdot[\beta\pm1.96\cdot se(\beta)]$ (8)

- Relative change in *Y* for an absolute change of *c* units in *X*

If (5) is the absolute change in *Y*, according to (3) the relative change in Y is:

$1+\frac{c\cdot\beta}{E\left[ Y \right]}$ (3)

For the 95% CI, from (6) and (3):

$1+\left\{ \frac{c\cdot\beta}{E[Y]}\pm1.96\cdot\frac{c\cdot se(\beta)}{E[Y]} \right\}$ (9)

That can be expressed as:

$\frac{c}{E[Y]}\left\{ \frac{E[Y]}{c}+\beta\pm1.96\cdot se(\beta) \right\}$ (10)

- Relative change in *Y* for a relative change of *k* times in *X*

Replacing *c* in (3) and (10) in accordance with (1):

$1+\frac{(k-1)\cdot E[X]\cdot\beta}{E[Y]}$ (11)

$\frac{(k-1)\cdot E[X]}{E[Y]}\left\{ \frac{E[Y]}{(k-1)\cdot E[X]}+\beta\pm1.96\cdot se(\beta) \right\}$ (12)

**Model B**

- Absolute change in *Y* for a relative change of *k* times in *X*

Directly from the model, the expression of effect size and the 95% CI are provided for (13) and (14):

$\log_{b} (k)\cdot\beta$ (13)

$\log_{b} (k)\cdot[\beta\pm1.96\cdot se(\beta)]$ (14)

- Absolute change in *Y* for an absolute change of *c* units in *X*

Replacing *k* in (13) and (14) in accordance with (2):

$\log_{b} (1+\frac{c}{E\left[ X \right]})\cdot\beta$ (15)

$\log_{b} (1+\frac{c}{E\left[ X \right]})\cdot[\beta\pm1.96\cdot se(\beta)]$ (16)

- Relative change in *Y* for a relative change of *k* times in *X*

If (13) is the absolute change in *Y*, according to (3) the relative change in *Y* is:

$1+\frac{\log_{b} (k)\cdot\beta}{E[Y]}$ (17)

For the 95% CI, from (14) and (3):

$1+\left\{ \frac{\log_{b} (k)\cdot\beta}{E[Y]}\pm1.96\cdot\frac{\log_{b} (k)\cdot se(\beta)}{E[Y]} \right\}$ (18)

That can be expressed as:

$\frac{\log_{b} (k)}{E[Y]}\left\{ \frac{E[Y]}{\log_{b} (k)}+\beta\pm1.96\cdot se(\beta) \right\}$ (19)

- Relative change in *Y* for an absolute change of *c* units in *X*

Replacing *k* in (17) and (19) in accordance with (2):

$1+\frac{\log_{b} (1+\frac{c}{E\left[ X \right]})\cdot\beta}{E[Y]}$ (20)

$\frac{\log_{b} (1+\frac{c}{E\left[ X \right]})}{E[Y]}\left\{ \frac{E[Y]}{\log_{b} (1+\frac{c}{E\left[ X \right]})}+\beta\pm1.96\cdot se(\beta) \right\}$ (21)

**Model C**

- Relative change in *Y* for an absolute change of *c* units in *X*

Directly from the model, the expression of effect size and the 95% CI are provided for (22) and (23):

$a^{c\cdot\beta}$ (22)

$a^{c\cdot[\beta\pm1.96\cdot se(\beta)]}$ (23)

- Relative change in *Y* for a relative change of *k* times in *X*

Replacing *c* in (22) and (23) in accordance with (1):

$a^{(k-1)\cdot E[X]\cdot\beta}$ (24)

$a^{(k-1)\cdot E[X]\cdot[\beta\pm1.96\cdot se(\beta)]}$ (25)

- Absolute change in *Y* for an absolute change of *c* units in *X*

If (22) is the relative change in *Y*, according to (4) the absolute change in *Y* is:

$(a^{c\cdot\beta}-1)\cdot E[Y]$ (4)

For the 95% CI, from (23) and (4):

${(a}^{c\cdot[\beta\pm1.96\cdot se(\beta)]}-1)\cdot E[Y]$ (26)

- Absolute change in *Y* for a relative change of *k* times in *X*

Replacing *c* in (4) and (26) in accordance with (1):

$(a^{(k-1)\cdot E[X]\cdot\beta}-1)\cdot E[Y]$ (27)

${(a}^{(k-1)\cdot E[X]\cdot[\beta\pm1.96\cdot se(\beta)]}-1)\cdot E[Y]$ (28)

**Model D**

- Relative change in *Y* for a relative change of *k* times in *X*

Directly from the model, the expression of effect size and the 95% CI are provided for (29) and (30):

$a^{\log_{b} (k)\cdot\beta}$ (29)

$a^{\log_{b} (k)\cdot[\beta\pm1.96\cdot se(\beta)]}$ (30)

- Relative change in *Y* for an absolute change of *c* units in *X*

Replacing *k* in (29) and (30) in accordance with (2):

$a^{\log_{b} (1+\frac{c}{E\left[ X \right]})\cdot\beta}$ (31)

$a^{\log_{b} (1+\frac{c}{E\left[ X \right]})\cdot[\beta\pm1.96\cdot se(\beta)]}$ (32)

- Absolute change in *Y* for a relative change of *k* times in *X*

If (29) is the relative change in *Y*, according to (4) the absolute change in *Y* is:

$${(a}^{\log_{b} (k)\cdot\beta}-1)\cdot E[Y]$$

(33)

For the 95% CI, from (30) and (4):

${(a}^{\log_{b} (k)\cdot[\beta\pm1.96\cdot se(\beta)]}-1)\cdot E[Y]$ (34)

- Absolute change in Y for an absolute change of c units in X

Replacing *k* in (33) and (34) in accordance with (2):

${(a}^{\log_{b} (1+\frac{c}{E\left[ X \right]})\cdot\beta}-1)\cdot E[Y]$ (35)

${(a}^{\log_{b} (1+\frac{c}{E\left[ X \right]})\cdot[\beta\pm1.96\cdot se(\beta)]}-1)\cdot E[Y]$ (36)
